# Trends in US Pediatric Unintentional Nonprescription Cold and Cough, Analgesic and Antipyretic Drug Exposure Cases amid the COVID-19 Pandemic

**DOI:** 10.1007/s40261-025-01444-9

**Published:** 2025-05-28

**Authors:** Sara Karami, Christian Angelo I. Ventura, Ellen Pinnow, Jody Green, Ajoa Asonye, Ibrahim T. Ibrahim, Lynda McCulley, Gerald J Dal Pan, Esther H. Zhou

**Affiliations:** Office of Surveillance and Epidemiology, Center for Drug Evaluation and Research (CDER), US Food and Drug Administration (FDA), 10903 New Hampshire Avenue, Building 22, Room 4202, Silver Spring, MD 20993 USA

## Abstract

**Background:**

The coronavirus disease 2019 (COVID-19) pandemic dramatically impacted healthcare systems.

**Objective:**

We assessed monthly unintentional pediatric (< 18 years) exposure case rate trends involving selected nonprescription cold and cough (CC), as well as analgesic and antipyretic (AA) drugs, before and during the COVID-19 pandemic, using the National Poison Data System (extracted August 2023).

**Methods:**

We included dextromethorphan, guaifenesin, phenylephrine, and pseudoephedrine CC drugs, and acetaminophen, naproxen, ibuprofen, and acetylsalicylic acid AA drugs; statins served as a control. We performed descriptive analyses involving single-product unintentional pediatric exposure cases overall, by sex, and by age. We performed interrupted time series (ITS) analyses, modeling associations between the pandemic’s immediate and sustained effects, adjusting for population and seasonality.

**Results:**

Overall, apart from the control, acetylsalicylic acid, and naproxen drugs, monthly unintentional single-product exposure case rates decreased sharply at the pandemic’s onset. In ITS analyses, rates decreased most notably for cases involving children < 6 years old, where unintentional-general and unintentional-therapeutic error case rates statistically significantly fell by 1.8–12.6 cases per million population at the pandemic’s onset. During the pandemic, case rates gradually increased to pre-pandemic levels within 1.5 years. For cases involving children < 6 years old, these exposure case rates statistically significantly rose by 0.1–0.6 cases per million population per month compared with pre-pandemic levels. Monthly case rate patterns for cases 6–12 years old mirrored those of cases < 6 years old, with less pronounced level and trend changes.

**Conclusions:**

These findings underscore the need for continuously adapting public health strategies to ensure drug safety during prolonged periods of public health emergencies.

**Supplementary Information:**

The online version contains supplementary material available at 10.1007/s40261-025-01444-9.

## Key Points

**Table:** 

The study revealed that pediatric unintentional exposures to nonprescription cold and cough (CC), and analgesic and antipyretic (AA) drugs decreased markedly with the onset of the COVID-19 pandemic.
Over time, CC and AA drug exposure cases rebounded, particularly increasing for unintentional-therapeutic errors among younger children toward the end of the pandemic.
These patterns highlight the importance of continuously adapting public health strategies to ensure drug safety during prolonged periods of a public health emergency.

## Introduction

The rapid spread of the highly contagious novel coronavirus 2 (SARS-CoV-2) and associated health complications at the COVID-19 pandemic’s onset triggered widespread disruptions. With nationwide lockdowns in place, acute respiratory infections dramatically decreased as people sheltered in place and avoided exposure to airborne pathogens. As a result, sales of many nonprescription products, especially cold and cough (CC) medicines, were speculated to decline at the beginning of the pandemic [1], although contradictory reports also suggested that panic-buying and widespread stockpiling of common nonprescription CC products early during the pandemic may have been a problem [2, 3]. Information from the World Health Organization (WHO) on COVID-19 management reported that most COVID-19-infected individuals developed mild-to-moderate symptoms and that individuals could recover without hospitalization [4]. People with mild-to-moderate COVID-19 symptoms may have managed symptoms at home with readily available nonprescription products, such as CC and analgesic and antipyretic (AA) drugs. Pediatric administration of these drugs requires careful attention, as children are particularly vulnerable to adverse effects of these medications when used improperly given the level of maturity of body systems involved in drug absorption, metabolism, transport, and elimination [5].

Comparing the pandemic with the pre-pandemic period, children may have been at a higher risk of unintentional exposures due to increased time spent at home during lockdowns, potentially leading to accidental ingestions facilitated by household organization and improper storage of medications. The aim of this study is to evaluate whether the COVID-19 pandemic impacted trends of unintentional pediatric United States (US) poison center (PC) exposure cases involving commonly used nonprescription CC and AA preparations.

## Methods

America’s Poison Centers’ National Poison Data System (NPDS) receives near real-time data from the US’s 55 PCs, which provide advice and information services and assistance to callers regarding exposures and non-exposures to prescription and nonprescription drugs and other substances*.* Each exposure case is assigned an exposure reason (e.g., intentional-abuse), a generic code (denoting a wide group of related products), and a unique product code if a specific substance is described [6]. Utilizing Micromedex^®^ Solutions, we mapped product and generic codes to commonly used CC drugs containing dextromethorphan, guaifenesin, phenylephrine, and pseudoephedrine, as well as AA drugs containing acetaminophen, naproxen, ibuprofen, and acetylsalicylic acid [7, 8], and segregated codes into three distinct typologies: nonprescription, prescription, or not otherwise specified. Two analysts independently evaluated these typologies; discrepancies (< 0.1% of total cases) were reconciled via a third analyst. We pre-defined HMG-CoA reductase inhibitors (statins) to serve as a control, assuming that long-term chronic use of this prescription drug would be minimally impacted by the pandemic [9, 10]. COVID-19 treatment guidelines recommended that patients continue using their medications and specifically mentioned statins [11].

Using identified product and generic codes, we extracted closed (i.e., follow-up related to exposures was complete) human unintentional exposure data from NPDS from 1 January 2015 through 5 May 2023 (end of the WHO’s pandemic declaration) [12]. The majority of unintentional PC exposures in children include unintentional-general and unintentional-therapeutic errors; other unintentional exposure categories typically occur less frequently and include unintentional-misuse and unintentional-unknown [6]. We excluded cases with patients aged ≥ 18 years, cases where initial calls were made from outside US states or territories, and cases with a medical outcome of confirmed non-exposure. Our study focused on single-product (i.e., a single-product comprised of single or multiple ingredients associated with exposure and where no other products were reported) unintentional exposure cases.

### Statistical Analyses

We performed descriptive analyses for unintentional CC, AA, and control single-product exposure cases. We assessed case counts and the percentage of cases involving unintentional single-product CC, AA, and control exposures for select characteristics for the entire study period. We generated monthly time-series plots for unintentional-general and unintentional-therapeutic error exposure cases, with case frequencies tabulated among stratified subgroups to visualize case patterns. We also examined trends in monthly pediatric exposure case rates per million population calculated using age-specific population estimates prepared by the Census Bureau in collaboration with the National Center for Health Statistics (NCHS) [13, 14].

We performed a segmented regression analysis, a quasi-experimental interrupted time-series (ITS) design, to model the immediate and sustained impact of the pandemic. We fit ITS models for monthly pediatric single-product unintentional exposure rates per million population for selected age groups involving study drugs after visual inspection of trends over time. The baseline period (pre-pandemic) was from 1 January 2015 to 29 February 2020. We treated 1 April 2020 as the start and 30 April 2023 as the end of the COVID-19 period, because April 2020 and April 2023 were the first and the last full months of the WHO’s pandemic declaration. We tested the immediate absolute change in monthly case rates immediately following the announcement of the pandemic (level change); we also evaluated the trend change during the pandemic period compared with the pre-pandemic period (trend change). We first used the X-12-ARIMA to remove seasonal variations where appropriate and then employed an autoregressive model to address remaining autocorrelation (correlation between exposure rates at different month lags) that might still exist before and during the pandemic [15–17]. We considered *p*-values < 0.01 from ITS analyses to be statistically significant. We used SAS to perform computational operations [18].

Lastly, to understand how COVID-19 disruptions may have influenced utilization of the nonprescription CC and AA products, we performed *a priori* descriptive analyses of monthly sales of these nonprescription products using the Consumer Health Insights and Private Label Ingredient Level Report (PL-ILR) databases. We evaluated trends in monthly nationally estimated sales of nonprescription (bottles/packages) products containing the study CC and AA drugs from June 2017 (the earliest available data) through April 2023. We used the IQVIA US Launch Edition to obtain monthly nationally estimated number of bottles/packages for statins sold from US outpatient pharmacies from June 2017 through April 2023.

This study was classified as a public health surveillance activity by the US Food and Drug Administration (FDA) Institutional Review Board.

## Results

### Exposure Case Characteristics

Table 1 depicts selected pediatric case characteristics for calls received by US PCs involving unintentional exposure to specific nonprescription CC (range: *N* = 22,960–73,559) and AA (*N* = 28,143–186,954) containing drugs and the control (statins: *N* = 23,093). Across study drugs, unintentional exposure cases most commonly involved a single product (CC: 84.9–86.8%, AA: 84.2–94.2%, and control: 54.5%). Across all single-product exposure cases, unintentional-general (CC: 52.4–61.4%, AA: 68.3–90.2%, and control: 90.8%) was a more common exposure reason than unintentional-therapeutic errors (CC: 37.3–46.6%, AA: 8.1–30.8%, and control: 8.9%). Among all single-product drugs examined, most unintentional exposure cases involved children < 6 years old (CC: 77.2–82.9%, AA: 88.4–92.7%, and control: 89.8%). Across study drugs, approximately half of the cases involved males (50.1–55.1%). The most common medical outcome involving single-product cases with a related clinical effect (1.7–9.8% across drugs) included minor effects (51.6–62.8% across drugs), followed by moderate (−1.4% to 14.8%) and major (0–1.1%) effects, with two deaths documented for acetaminophen.

**Table 1:** Selected pediatric case characteristics involving unintentional nonprescription cold and cough, analgesic and antipyretic drugs, and statins (the control), National Poison Data System, USA, 1 January 2015 to 5 May 2023

Characteristics	Cold and cough drugs				Analgesic and antipyretic drugs				Control
	Dextrometh-orphan	Guaifenesin	Phenylephrine	Pseudoephedrine	Acetaminophen	Naproxen	Ibuprofen	Acetylsalicylic acid	Statins
	*N* (%)^a^	*N* (%)^a^	*N* (%)^a^	*N* (%)^a^	*N* (%)^a^	*N* (%)^a^	*N* (%)^a^	*N* (%)^a^	*N* (%)^a^
**Type of exposures**									
Total	73,559 (100)	29,229 (100)	44,144 (100)	22,960 (100)	186,954 (100)	13,543 (100)	164,260 (100)	28,143 (100)	23,093 (100)
Multiple-product	9718 (13.2)	4427 (15.2)	6600 (15.0)	3369 (14.7)	18,670 (10.0)	1601 (11.8)	9462 (5.8)	4446 (15.8)	10,513 (45.5)
Single-product	63,841 (86.8)	24,802 (84.9)	37,544 (85.1)	19,591 (85.3)	168,284 (90.0)	11,942 (88.2)	154,798 (94.2)	23,697 (84.2)	12,580 (54.5)
**Single-product cases (below)**									
*Unintentional exposure reason*^b^									
General	33,431 (52.4)	13,380 (54.0)	23,063 (61.4)	11,920 (60.8)	115,003 (68.3)	10,619 (88.9)	106,099 (68.5)	21,362 (90.2)	11,420 (90.8)
Therapeutic error	29,721 (46.6)	11,144 (44.9)	14,012 (37.3)	7481 (38.2)	51,430 (30.6)	1135 (9.5)	47,672 (30.8)	1913 (8.1)	1119 (8.9)
Other^c^	689 (1.0)	278 (1.1)	469 (1.3)	190 (1.0)	1851 (1.1)	188 (1.6)	1027 (0.7)	422 (1.7)	41 (0.3)
*Age category*^b^									
< 6 years	49,260 (77.2)	19,244 (77.6)	31,127 (82.9)	15,805 (80.7)	148,803 (88.4)	10.261 (85.9)	143,511 (92.7)	20,972 (88.5)	11,290 (89.8)
6–12 years	11,953 (18.7)	4186 (16.9)	5336 (14.2)	2803 (14.3)	13,546 (8.1)	635 (5.3)	8950 (5.8)	1679 (7.1)	969 (7.7)
13–17 years	2628 (4.1)	1372 (5.5)	1081 (2.9)	983 (5.0)	5935 (3.5)	1046 (8.8)	2337 (1.5)	1046 (4.4)	321 (2.6)
*Sex*^b^									
Male	34,430 (53.9)	13,429 (54.1)	19,848 (52.9)	10,394 (53.1)	87,374 (51.9)	5988 (50.1)	82,148 (53.1)	12,040 (50.8)	6933 (55.1)
Female	29,325 (45.9)	11,346 (45.8)	17,627 (47.0)	9172 (46.8)	80,605 (47.9)	5944 (49.8)	72,405 (46.8)	11,608 (49.0)	5612 (44.6)
Unknown	86 (0.1)	27 (0.1)	69 (0.2)	25 (0.1)	305 (0.2)	10 (0.1)	245 (0.2)	49 (0.2)	35 (0.3)
**Related single-product cases (below)**									
*Related cases*^b,d^	6246 (9.8)	1454 (5.9)	2497 (6.7)	1560 (8.0)	5826 (3.5)	521 (4.4)	3124 (2.0)	1252 (5.3)	219 (1.7)
*Related medical outcomes*^e,f^									
Minor	3561 (57.0)	839 (57.7)	1477 (59.2)	979 (62.8)	3509 (60.2)	269 (51.6)	1716 (54.9)	670 (53.5)	114 (52.1)
Moderate	874 (14.0)	114 (7.8)	158 (6.3)	115 (7.4)	613 (10.5)	17 (3.3)	136 (4.4)	185 (14.8)	3 (1.4)
Major	49 (0.8)	9 (0.6)	15 (0.6)	13 (0.8)	61 (1.1)	0 (0)	17 (0.5)	6 (0.5)	0 (0)
Death	0 (0)	0 (0)	0 (0)	0 (0)	2 (0.03)	0 (0)	0 (0)	1 (0.1)	0 (0)
Not followed, minimal effect possible	1538 (24.6)	435 (29.9)	771 (30.9)	399 (25.6)	1393 (23.9)	217 (41.7)	1186 (38.0)	343 (27.4)	99 (45.2)
No followed, potentially toxic effect	220 (3.5)	56 (3.9)	74 (3.0)	53 (3.4)	239 (4.1)	17 (3.3)	64 (2.1)	45 (3.6)	3 (1.4)
Unrelated effect	3 (0.1)	1 (0.1)	2 (0.1)	1 (0.1)	8 (0.1)	1 (0.2)	4 (0.1)	2 (0.2)	0 (0)

^a^Percentages calculated among the total number of exposure cases for the respective drug, unless specified otherwise; percentages may not sum to 100 due to rounding

^b^Includes single-product cases only; percentages calculated among the number of single-product exposure cases for the respective drug; percentages may not sum to 100 due to rounding

^c^Not a National Poison Data System (NPDS) exposure reason category; includes NPDS unintentional exposure reason categories: bite/sting, environmental, food poisoning, misuse, occupational, and unknown

^d^Includes cases with at least one clinical effect deemed “related” to exposure. “Relatedness” refers to a level of precision for clinical effects that poison center specialists determine based on timing, severity, and assessment of clinical effects for reported exposure by poison center specialists. Excluded cases with clinical effects deemed “unrelated” or “unknown if related” to exposure: dextromethorphan *N* = 42,221; guaifenesin *N* = 17,320; phenylephrine *N* = 25,468; pseudoephedrine *N* = 12,901; acetaminophen *N* = 118,833; naproxen *N* = 8001; ibuprofen *N* = 118,517; acetylsalicylic acid *N* = 14,857; statins *N* = 9636. Excludes cases with *no effect*: dextromethorphan *N* = 15,374; guaifenesin *N* = 6028; phenylephrine *N* = 9579; pseudoephedrine *N* = 5130; acetaminophen *N* = 43,626; naproxen *N* = 3420; ibuprofen *N* = 33,157; acetylsalicylic acid *N* = 7588; statins *N* = 2725

^e^Medical outcomes also include acetaminophen cases with “related” *no effect* (*N* = 1), ibuprofen *not followed, judged as nontoxic exposure (clinical effect not expected* (*N* = 1), and dextromethorphan “related” *no effect* (*N* = 1). Minor effects include those involving some minimally bothersome symptoms. Moderate effects include those involving more pronounced or prolonged symptoms or symptoms of a more systemic nature than minor symptoms. Major effects include those involving life-threatening symptoms or significant residual disability/disfigurement

^f^Includes related single-product cases only; percentages calculated among the number of related single-product exposure cases for the respective drug; percentages may not sum to 100 due to rounding

### Monthly Unintentional Exposure Case Rates by Age Group

**Fig. 1:**
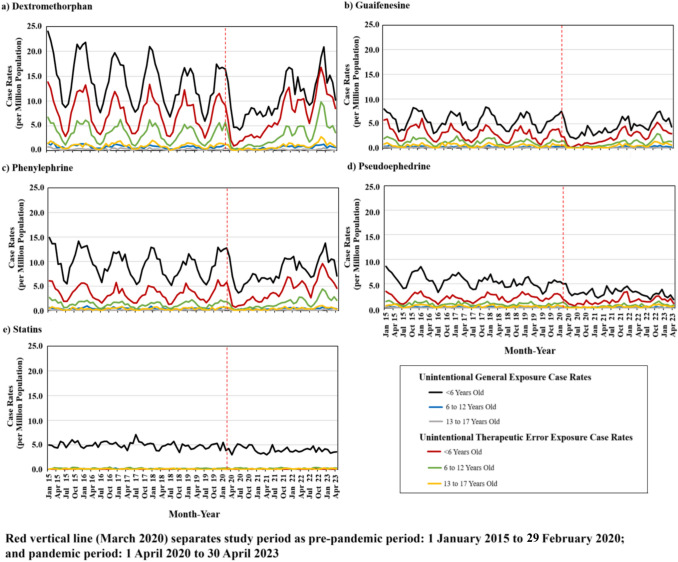
Monthly single-product unintentional-general and unintentional-therapeutic error exposure case rates involving selected nonprescription cold and cough drugs, and statins (the control), by age group

Figure 1 depicts trends in monthly single-product unintentional-general and unintentional-therapeutic error exposure case rates involving nonprescription CC drugs and the control, stratified by age group. During the pre-pandemic period, dextromethorphan, guaifenesin, and phenylephrine unintentional case rates gradually decreased with an expected seasonal pattern. At the pandemic’s onset, monthly case rates involving these drugs, for children < 6 years old, immediately and sharply declined, then eventually returned to pre-pandemic levels. For dextromethorphan and phenylephrine specifically, monthly case rates involving unintentional-therapeutic errors surpassed pre-pandemic case rates approximately 1.5 years into the pandemic. ITS analyses involving dextromethorphan, guaifenesin, and phenylephrine in Table 2, revealed that among children < 6 years old, case rates statistically significantly dropped by 4.9, 2.7, and 2.4 cases per million population, respectively, for unintentional-general exposures; and by 5.3, 1.8, and 2.0 cases per million population, respectively, for unintentional-therapeutic errors at the pandemic’s onset. During the pandemic, both unintentional-general and unintentional-therapeutic error case rates gradually and statistically significantly increased by 0.3, 0.1, and 0.2 cases per million population per month, for the three drugs, respectively.

**Table 2:** Interrupted time-series analysis on the association of the COVID-19 pandemic and pediatric exposure case rates per million population to the selected single-product nonprescription cold and cough, analgesic and antipyretic drugs, and statins (control), by age group, adjusted for seasonality, National Poison Data System, 1 January 2015 to 30 April 2023

	Children involving cases < 6 years old				Cases 6–12 years old				Cases 13–17 years old			
	Baseline level^a^ estimate (95% CI)	Trend pre-pandemic ^b^ (95% CI)	Level change^c^ (95% CI)	Trend change^d^ (95% CI)	Baseline level^a^ estimate (95% CI)	Trend pre-pandemic ^b^ (95% CI)	Level change^c^ (95% CI)	Trend change^d^ (95% CI)	Baseline level^a^ estimate (95% CI)	Trend pre-pandemic ^b^ (95% CI)	Level change^c^ (95% CI)	Trend change^d^ (95% CI)
**Unintentional-general exposure case rates**												
*Cold and cough drugs*												
Dextromethorphan	16.490 (15.968, 17.012)	− 0.085 (− 0.099, − 0.070)^e^	− 4.883 (− 5.841, − 3.925)^e^	0.320 (0.283, 0.357)^e^	0.973 (0.888, 1.058)	− 0.007 (− 0.009, − 0.005)^e^	− 0.320 (− 0.465, − 0.175)^e^	0.028 (0.022, 0.034)^e^	NR	NR	NR	NR
Guaifenesin	5.994 (5.783, 6.204)	− 0.012 (− 0.018, − 0.006)^e^	− 2.693 (− 3.074, − 2.312)^e^	0.106 (0.091, 0.120)^e^	0.325 (0.293, 0.357)	− 0.002 (− 0.003, − 0.001)^e^	− 0.144 (− 0.201, − 0.088)^e^	0.010 (0.008, 0.012)^e^	NR	NR	NR	NR
Phenylephrine	10.613 (10.208, 11.017)	− 0.039 (− 0.050, − 0.028)^e^	− 2.437 (− 3.179, − 1.695)^e^	0.163 (0.134, 0.192)^e^	0.455 (0.392, 0.517)	− 0.002 (− 0.004, 0.000)h	− 0.090 (− 0.192, 0.013)	0.010 (0.006, 0.014)^e^	NR	NR	NR	NR
*Analgesic and antipyretic drugs*												
Acetaminophen	54.559 (53.035, 56.083)	− 0.195 (− 0.237, − 0.153)^e^	− 7.192 (− 9.711, − 4.673)^e^	0.449 (0.347, 0.550)^e^	1.629 (1.538, 1.720)	− 0.007 (− 0.010, − 0.005)^e^	− 0.233 (− 0.398, − 0.067)^g^	0.028 (0.021, 0.034)^e^	0.682 (0.603, 0.761)	0.001 (− 0.002, 0.003)	− 0.157 (− 0.305, − 0.009)	0.009 (0.004, 0.015)^g^
Ibuprofen	57.654 (56.563, 58.745)	− 0.286 (− 0.317, − 0.256)^e^	− 12.586 (− 14.562, − 10.610)^e^	0.582 (0.505, 0.660)^e^	1.214 (1.141, 1.287)	− 0.006 (− 0.008, − 0.004)^e^	− 0.357 (− 0.489, − 0.225)^e^	0.017 (0.012, 0.022)^e^	0.412 (0.348, 0.477)	− 0.001 (− 0.003, 0.001)	− 0.061 (− 0.176, 0.054)	0.001 (− 0.004, 0.005)
Acetylsalicylic acid	10.966 (10.371, 11.562)	− 0.032 (− 0.049, − 0.016)^f^	− 2.393 (− 3.357, − 1.428)^e^	0.002 (− 0.037, 0.041)	0.282 (0.248, 0.315)	0.000 (− 0.001, 0.001)	− 0.112 (− 0.170, − 0.054)^f^	0.001 (− 0.001, 0.001)	0.189 (0.136, 0.241)	− 0.001 (− 0.002, 0.001)	− 0.018 (− 0.101, 0.065)	0.001 (− 0.002, 0.004)
*Control drug*												
Statins	5.286 (4.994, 5.579)	− 0.010 (− 0.018, − 0.002)	− 0.425 (− 0.863, 0.014)	− 0.002 (− 0.020, 0.017)	0.128 (0.096, 0.160)	− 0.001 (− 0.002, 0.001)	0.014 (− 0.039, 0.067)	− 0.0003 (− 0.003, 0.002)	0.023 (0.009, 0.036)	0.001 (− 0.0003, 0.001)	− 0.012 (− 0.036, 0.011)	− 0.0008 (− 0.001, 0.0008)
**Unintentional-therapeutic error exposure case rates**												
*Cold and cough drugs*												
Dextromethorphan	8.542 (8.039, 9.044)	− 0.032 (− 0.046, − 0.018)^e^	− 5.286 (− 6.230, − 4.343)^e^	0.338 (0.303, 0.374)^e^	4.138 (3.832, 4.444)	− 0.021 (− 0.029, − 0.012)^e^	− 2.677 (− 3.242, − 2.111)^e^	0.169 (0.15, 0.19)^e^	NR	NR	NR	NR
Guaifenesin	3.858 (3.469, 4.248)	− 0.026 (− 0.037, − 0.016)^e^	− 1.785 (− 2.299, − 1.270)^e^	0.121 (0.097, 0.145)^e^	1.547 (1.452, 1.641)	− 0.009 (− 0.011, − 0.006)^e^	− 0.876 (− 1.049, − 0.702)^e^	0.054 (0.047, 0.060)^e^	NR	NR	NR	NR
Phenylephrine	3.778 (3.3995, 4.161)	− 0.010 (− 0.020, 0.001)	− 2.021 (− 2.655, − 1.386)^e^	0.169 (0.142, 0.196)^e^	1.485 (1.313, 1.657)	− 0.006 (− 0.011, − 0.001)	− 0.902 (− 1.191, − 0.614)^e^	0.074 (0.063, 0.085)^e^	NR	NR	NR	NR
*Analgesic and antipyretic drugs*												
Acetaminophen	14.826 (13.636, 16.015)	0.041 (0.009, 0.074)	− 8.579 (− 10.463, − 6.694)^e^	0.318 (0.241, 0.395)^e^	3.086 (2.822, 3.350	− 0.001 (− 0.008, 0.007)	− 2.109 (− 2.585, − 1.633)^e^	0.115 (0.097, 0.133)^e^	1.579 (1.459, 1.699)	0.002 (− 0.001, 0.005)	− 0.611 (− 0.828, − 0.394)^e^	0.038 (0.03, 0.046)^e^
Ibuprofen	18.736 (18.023, 19.450)	− 0.017 (− 0.037, 0.003)	− 10.241 (− 11.576, − 8.906)^e^	0.363 (0.312, 0.414)^e^	2.925 (2.656, 3.195)	− 0.014 (− 0.022, − 0.007)^f^	− 1.863 (− 2.282, − 1.445)^e^	0.083 (0.064, 0.101)^e^	0.710 (0.619, 0.801)	− 0.001 (− 0.004, 0.002)	− 0.288 (− 0.438, − 0.138)^f^	0.012 (0.006, 0.018)^e^
Acetylsalicylic acid	0.168 (0.108, 0.229)	0.003 (0.001, 0.004)^g^	− 0.225 (− 0.307, − 0.143)^e^	− 0.0003 (− 0.004, 0.003)	0.284 (0.229, 0.339)	0.001 (− 0.001, 0.003)	− 0.294 (− 0.385, − 0.204)^e^	0.009 (0.006, 0.013)^e^	0.269 (0.220, 0.319)	0.0003 (− 0.001, 0.002)	− 0.102 (− 0.187, − 0.017)	0.002 (− 0.001, 0.005)
*Control drug*												
Statins	0.102 (0.074, 0.130)	− 0.001 (− 0.001, 0.0003)	− 0.040 (− 0.086, 0.006)	0.001 (− 0.0004, 0.003)	0.256 (0.232, 0.280)	− 0.001 (− 0.002, − 0.0002)	0.015 (− 0.028, 0.058)	0.002 (0.001, 0.004)	0.139 (0.108, 0.169)	− 0.001 (− 0.002, 0.002)	− 0.006 (− 0.060, 0.049)	0.004 (0.002, 0.006)^f^

Note: We did not conduct the ITS analysis for pseudoephedrine and naproxen, as the visual trends remained stable throughout the study period. Period before the pandemic: 1 January 2015 to 29 February 2020. Period after onset of the pandemic: 1 April 2020 to 30 April 2023. Trend after the onset of the pandemic = trend before the pandemic + trend change. An estimate > 0 indicates an increasing trend, while < 0 indicates a decreasing trend

*CI* confidence interval, *NR* not reported (due to small and unstable estimates)

^a^Baseline level: average outcome at baseline (time 0).

^b^Trend before the pandemic: the slope of the ITS trajectory before the pandemic. An estimate > 0 indicates an increasing trend, while < 0 indicates a decreasing trend

^c^Level change: the difference in average outcome compared right after the onset of the pandemic to right before the pandemic

^d^Trend change: the difference in slopes of the trend compared after the onset of the pandemic to before the pandemic

^e^*p*-value < 0.0001

^f^*p*-value < 0.001

^g^*p*-value <0.01

For cases of children 6–12 years of age involving the CC drugs, the patterns were notably parallel to patients involving children < 6 years old, although level and trend changes were less pronounced (Fig. 1). Specifically, at the pandemic’s onset, case rates for unintentional-general exposures involving dextromethorphan and guaifenesin statistically significantly declined, and there was a small, but statistically significant increase during the pandemic. For phenylephrine, no statistically significant decrease was noted for unintentional-general exposure case rates, although a small and statistically significant increased trend change was observed. For unintentional-therapeutic errors, dextromethorphan, guaifenesin, and phenylephrine case rates statistically significantly decreased at the pandemic’s onset by 2.7, 0.9, and 0.9 cases per million population, respectively, and then increased significantly during the pandemic by 0.2, 0.1, and 0.1 cases per million population per month, respectively (Table 2).

For CC drug unintentional exposures in patients 13–17 years old, monthly case rates were low, and ITS analyses were not done owing to unstable estimates. Nevertheless, we noted for patients 13–17 years involving pseudoephedrine, unintentional exposure case rates followed a similar trend of declining seasonal patterns before the pandemic, with a noticeable drop immediately after the pandemic onset (Fig. 1). Our control substance, statins, showed no noticeable impact from the COVID-19 pandemic (Fig. 1; Table 2).

**Fig. 2:**
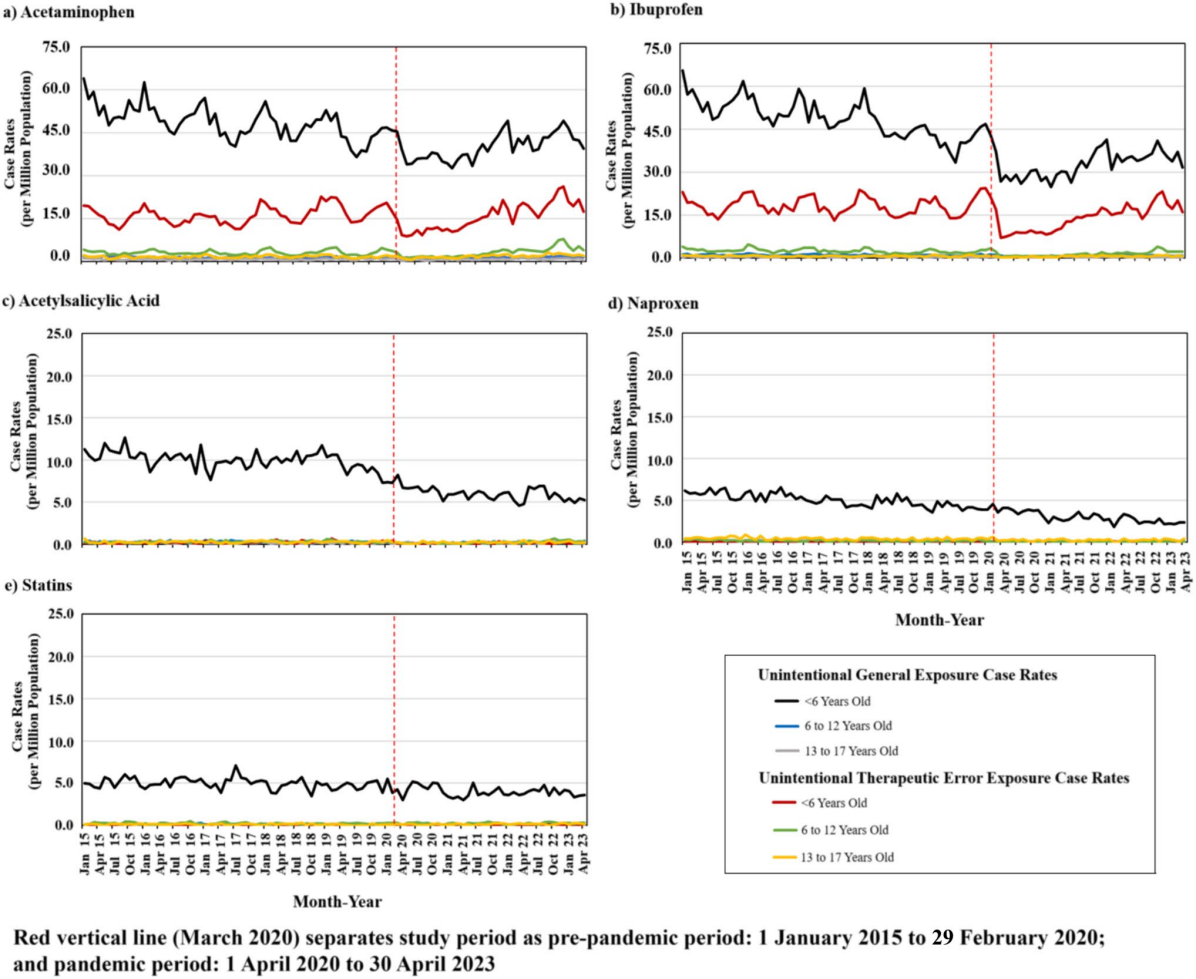
Monthly single-product unintentional-general and unintentional-therapeutic error exposure case rates involving selected nonprescription analgesic and antipyretic drugs, and statins (the control), by age group

Figure 2 depicts trends in monthly single-product unintentional-general and unintentional-therapeutic error exposure case rates involving nonprescription AA drugs (and control) by age group. During the pre-pandemic period, acetaminophen and ibuprofen unintentional exposure case rates gradually decreased with an expected seasonal pattern, followed by an immediate drop in monthly case rates at the pandemic’s onset, followed by a subsequent increase in case rates to near pre-pandemic levels approximately 1 year later. ITS analysis (Table 2) revealed that for children < 6 years old, acetaminophen, ibuprofen, and acetylsalicylic acid unintentional-general case rates statistically significantly decreased by 7.2, 12.6, and 2.4 cases per million population, respectively, immediately after the pandemic’s onset. Yet, during the pandemic, there was a gradual and statistically significant monthly increase compared with the pre-pandemic period for acetaminophen and ibuprofen drugs. For unintentional-therapeutic errors involving children < 6 years old with acetaminophen, ibuprofen, and acetylsalicylic acid exposures, there was a statistically significant initial drop in case rates. During the pandemic, acetaminophen and ibuprofen case rates statistically significantly increased.

For patients 6–12 years old, acetaminophen, ibuprofen, and acetylsalicylic acid unintentional exposure case rate patterns were notably parallel to patients involving children < 6 years old, although the level and trend changes were less pronounced. Specifically, at the onset of the pandemic, there was a statistically significant drop in acetaminophen, ibuprofen, and acetylsalicylic acid case rates for both unintentional-general and unintentional-therapeutic error. During the pandemic, there was a small but statistically significant increase in case rates involving acetaminophen and ibuprofen unintentional-general, as well as acetaminophen, ibuprofen, and acetylsalicylic acid unintentional-therapeutic error. Near the end of the pandemic (fourth quarter of 2022), acetaminophen unintentional-therapeutic error exposure monthly case rates surpassed pre-pandemic levels (Fig. 2; Table 2).

For patients 13–17 years old, unintentional exposure case rates were small, and no statistically significant level or trend changes were observed, apart from unintentional-therapeutic errors involving acetaminophen and ibuprofen. Specifically, for these two AA drugs, unintentional-therapeutic error case rates statistically significantly dropped at the pandemic’s onset and then increased statistically significantly during the pandemic. Trends in monthly naproxen unintentional single-product exposure case rates typically decreased across both the pre-pandemic and pandemic periods, while the trend appeared relatively steady for the control medication, statins, as aforementioned (Fig. 2; Table 2).

### Monthly Unintentional Exposure Case Rates by Sex

**Fig. 3:**
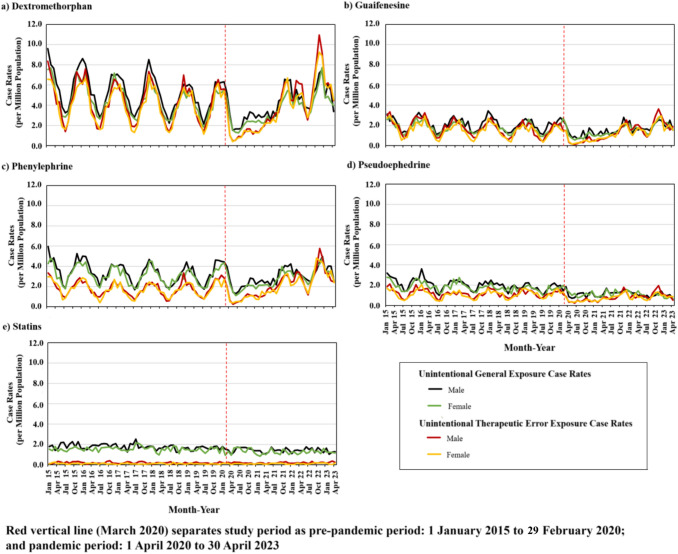
Monthly single-product unintentional-general and unintentional-therapeutic error exposure case rates involving selected nonprescription cold and cough drugs, and statins (the control), by sex

**Fig. 4:**
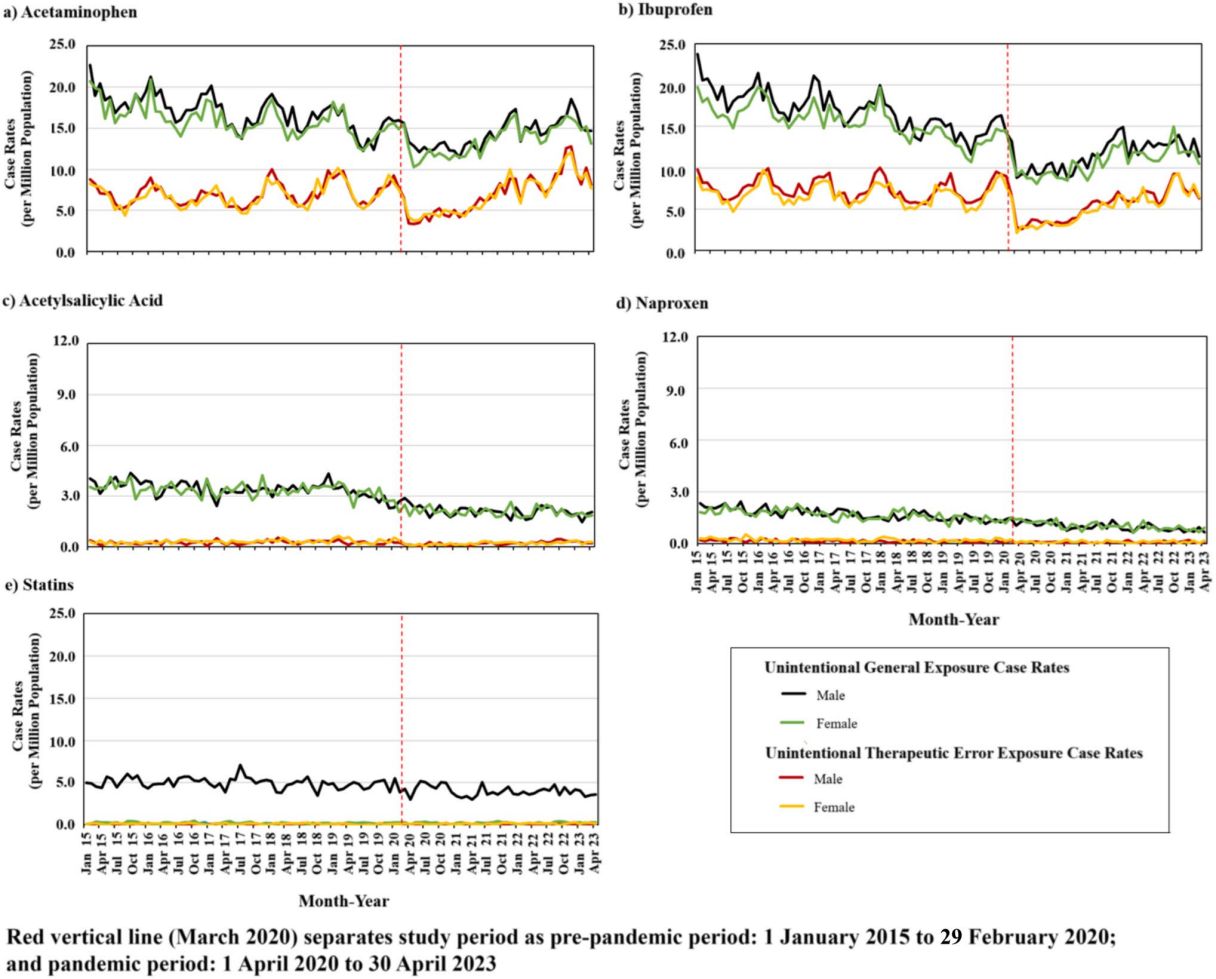
Monthly single-product unintentional-general and unintentional-therapeutic error exposure case rates involving selected nonprescription analgesic and antipyretic drugs, and statins (the control), by sex

Sex stratified monthly exposure case rates involving single-product unintentional CC (Fig. 3) and AA (Fig. 4) drugs and the control did not show any meaningful differences by sex. Overall, during the pre-pandemic period unintentional CC and AA exposure case rates gradually decreased with an expected seasonal pattern, apart from acetylsalicylic acid and naproxen, where seasonal effects were not observed. At the onset of the pandemic, case rates immediately and sharply declined for both sexes, although less noticeably for acetylsalicylic acid and naproxen. During the pandemic, case rates eventually returned to pre-pandemic levels, except for acetylsalicylic acid and naproxen where rates continued to marginally decrease.

ITS analysis involving dextromethorphan, guaifenesin, and phenylephrine CC drugs, and acetaminophen, ibuprofen, and acetylsalicylic acid AA drugs, revealed that case rates at the pandemic’s onset statistically significantly dropped for both unintentional-general and unintentional-therapeutic error exposures, regardless of sex (Table 3). During the pandemic, case rates involving these drugs were small yet statistically significantly increased for unintentional-general and unintentional-therapeutic error exposures, regardless of sex, except for acetylsalicylic acid and naproxen exposures where the trends had been decreasing throughout the study period. Near the end of the pandemic (fourth quarter of 2022), male and female unintentional-therapeutic error case rates involving dextromethorphan, guaifenesin, and phenylephrine CC drugs, and involving acetaminophen, surpassed pre-pandemic case rates. Trends involving the control medication, statins, appeared relatively steady across the entire study period for both male and female case rates; no statistically significant level changes were noted (Figs. 3, 4; Table 3).

**Table 3:** Interrupted time-series analysis on the association of the COVID-19 pandemic and pediatric exposure case rates per million population to select single-product nonprescription cold and cough, analgesic and antipyretic drugs, and statins (the control), by sex, adjusted for seasonality, National Poison Data System, 1 January 2015 to 30 April 2023

	Male cases				Female cases			
	Baseline level^a^ estimate (95% CI)	Trend pre-pandemic ^b^ (95% CI)	Level change^c^ (95% CI)	Trend change^d^ (95% CI)	Baseline level^a^ estimate (95% CI)	Trend pre-pandemic ^b^ (95% CI)	Level change^c^ (95% CI)	Trend change^d^ (95% CI)
**Unintentional-general exposure case rates**								
*Cold and cough drugs*								
Dextromethorphan	6.274 (6.061, 6.487)	− 0.034 (− 0.04, − 0.028)^e^	− 1.860 (− 2.258, − 1.462)^e^	0.128 (0.113, 0.143)^e^	5.376 (5.183, 5.569)	− 0.029 (− 0.035, − 0.024)^e^	− 1.713 (− 2.077, − 1.350)^e^	0.122 (0.108, 0.136)^e^
Guaifenesin	2.261 (2.179, 2.343)	− 0.005 (− 0.007, − 0.003)^e^	− 1.008 (− 1.155, − 0.861)^e^	0.039 (0.033, 0.045)^e^	1.961 (1.852, 2.069)	− 0.005 (− 0.008, − 0.002)^f^	− 0.889 (− 1.067, − 0.711)^e^	0.043 (0.036, 0.050)^e^
Phenylephrine	3.882 (3.694, 4.069)	− 0.016 (− 0.022, − 0.011)^e^	− 0.696 (− 1.039, − 0.354)^e^	0.060 (0.047, 0.073)^e^	3.462 (3.311, 3.613)	− 0.012 (− 0.016, − 0.008)^e^	− 0.965 (− 1.239, − 0.690)^e^	0.064 (0.053, 0.074)^e^
*Analgesic and antipyretic drugs*								
Acetaminophen	19.275 (18.811, 19.7438)	− 0.075 (− 0.088, − 0.062)^e^	− 2.358 (− 3.185, − 1.532)^e^	0.184 (0.152, 0.215)^e^	18.053 (17.316, 18.791)	− 0.066 (− 0.086, − 0.046)^e^	− 2.343 (− 3.426, − 1.261)^e^	0.170 (0.123, 0.217)^e^
Ibuprofen	20.339 (19.975, 20.704)	− 0.108 (− 0.118, − 0.098)^e^	− 4.184 (− 4.852, − 3.516)^e^	0.227 (0.201, 0.253)^e^	18.304 (17.862, 18.746)	− 0.086 (− 0.099, − 0.074)^e^	− 4.227 (− 5.046, − 3.408)^e^	0.192 (0.161, 0.223)^e^
Acetylsalicylic acid	3.828 (3.597, 4.060)	− 0.013 (− 0.020, − 0.007)^e^	− 0.737 (− 1.113, − 0.361)^f^	0.005 (− 0.010, 0.020)	3.620 (3.443, 3.795)	− 0.009 (− 0.013, − 0.004)^f^	− 0.944 (− 1.233 − 0.654)^e^	0.005 (− 0.007, 0.016)
*Control drug*								
Statins	1.953 (1.874, 2.033)	− 0.006 (− 0.008, − 0.003)^e^	− 0.169 (− 0.313, − 0.025)	0.006 (0.000, 0.011)	1.632 (1.529, 1.734)	− 0.003 (− 0.005, 0.000)	− 0.163 (− 0.332, 0.006)	0.001 (− 0.005, 0.008)
**Unintentional-therapeutic error exposure case rates**								
*Cold and cough drugs*								
Dextromethorphan	4.943 (4.649, 5.236)	− 0.022 (− 0.030, − 0.014)^e^	− 3.048 (− 3.597, − 2.499)^e^	0.200 (0.179, 0.221)^e^	4.441 (4.205, 4.678)	− 0.016 (− 0.023, − 0.010)^e^	− 2.979 (− 3.423, − 2.535)^e^	0.182 (0.166, 0.199)^e^
Guaifenesin	2.240 (2.09, 2.389)	− 0.016 (− 0.020, − 0.012)^e^	− 1.063 (− 1.322, − 0.804)^e^	0.076 (0.066, 0.086)^e^	1.813 (1.686, 1.940)	− 0.010 (− 0.013, − 0.006)^e^	− 0.932 (− 1.151, − 0.712)^e^	0.061 (0.052, 0.069)^e^
Phenylephrine	1.958 (1.772, 2.143)	− 0.006 (− 0.011, − 0.001)	− 1.031 (− 1.366, − 0.697)^e^	0.090 (0.077, 0.103)^e^	1.885 (1.700, 2.069)	− 0.006 (− 0.011, − 0.001)	− 1.128 (− 1.415, − 0.840)^e^	0.093 (0.080, 0.105)^e^
*Analgesic and antipyretic drugs*								
Acetaminophen	6.676 (6.082, 7.270)	0.010 (− 0.006, 0.026)	− 3.886 (− 4.703, − 3.068)^e^	0.178 (0.141, 0.215)^e^	6.309 (5.794, 6.824)	0.015 (0.001, 0.029)	− 3.573 (− 4.400, − 2.745)^e^	0.154 (0.120, 0.187)^e^
Ibuprofen	7.891 (7.568, 8.213)	− 0.016 (− 0.025, − 0.007)^f^	− 4.03 (− 4.634, − 3.426)^e^	0.158 (0.135, 0.181)^e^	7.036 (6.798, 7.275)	− 0.010 (− 0.017, − 0.003)^g^	− 4.167 (− 4.616, − 3.718)^e^	0.166 (0.149, 0.183)^e^
Acetylsalicylic acid	0.233 (0.186, 0.280)	0.001 (− 0.0004, 0.002)	− 0.203 (− 0.279, − 0.126)^e^	0.006 (0.003, 0.009)^f^	0.251 (0.211, 0.291)	0.002 (0.001, 0.003)^f^	− 0.249 (− 0.317, − 0.181)^e^	0.003 (− 0.0001, 0.005)
*Control drug*								
Statins	0.220 (0.187, 0.253)	− 0.001 (− 0.002, 0.0001)	− 0.016 (− 0.074, 0.042)	0.003 (0.001, 0.005)	0.119 (0.100, 0.137)	− 0.0004 (− 0.001, 0.0001)	− 0.011 (− 0.044, 0.022)	0.002 (0.001, 0.003)^g^

Note: we did not conduct the ITS analysis for pseudoephedrine and naproxen, as the visual trends remained stable throughout the study period. Period before the pandemic: 1 January 2015 to 29 February 2020. Period after onset of the pandemic: 1 April 2020 to 30 April 2023. Trend after the onset of the pandemic = trend before the pandemic + trend change. An estimate > 0 indicates an increasing trend, while < 0 indicates a decreasing trend

*CI* confidence interval

^a^Baseline level: average outcome at baseline (time 0)

^b^Trend before the pandemic: the slope of the ITS trajectory before the pandemic. An estimate > 0 indicates an increasing trend, while < 0 indicates a decreasing trend

^c^Level change: the difference in average outcome compared right after the onset of the pandemic to right before the pandemic

^d^Trend change: the difference in slopes of the trend compared after the onset of the pandemic to before the pandemic

^e^*p*-value < 0.0001

^f^*p*-value < 0.001

^g^*p*-value < 0.01

Trends in monthly single-product unintentional exposure case counts revealed relatively similar patterns to those described above for monthly case rates (Supplemental Figs. 1 and 2).

### Trends in Monthly Estimated Sales

**Fig. 5:**
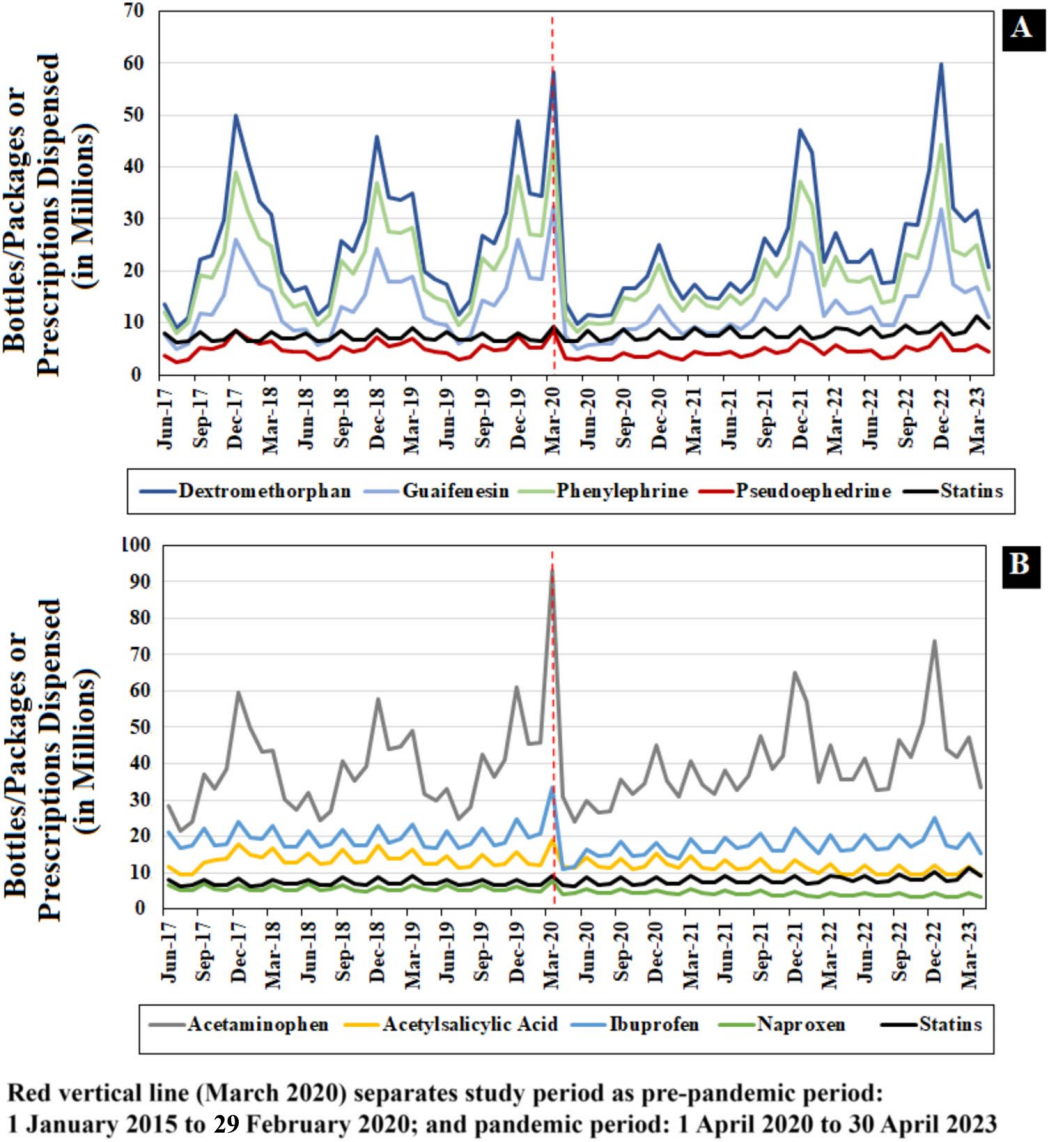
National monthly estimates* of selected over-the-counter sales of cold and cough (**A**), and analgesic and antipyretic (**B**) products from retail stores to consumers; and national monthly estimates of statins (panels **A** and **B**) from US outpatient pharmacies to consumers, June 2017 through April 2023. Source: Nonprescription International Market Tracking, Private Label Ingredient Level Report and IQVIA US Launch Edition, 2017–2023. Data extracted August 2024. *The IQVIA OTC data includes only retail sales data (from retail drug pharmacies, mass merchandisers, grocery stores, and military commissaries. Retail sales data do not capture sales activity from Costco, convenience stores, specialty stores, internet sales, phone sales, or kiosks

*A priori* descriptive drug utilization analyses, illustrated in Fig. 5, generally showed that sales of nonprescription CC and AA products from retail stores to consumers gradually increased from August through March of each examined year (2017–2022) before experiencing a steady decline during all other months. Overall, the largest increase was observed in the month of December (2017–2022), except for 2020, when the biggest spike in sales was recorded in March. The sales of nonprescription products examined were highest in the fourth quarter of 2019 and the first quarter of 2020 before decreasing thereafter. Peaks were also observed in the fourth and first quarters of 2021–2022 and 2022–2023, respectively. For statins, the estimated monthly number of bottles/units sold from outpatient US pharmacies remained relatively steady but increased marginally from June 2019 through April 2023. Both the seasonal patterns and overall trends of the drug utilization data mirror the single-product unintentional exposure cases from June 2017 to April 2023.

## Discussion

Our findings highlight pronounced fluctuations in pediatric exposure case rates involving select nonprescription CC and AA drugs during the COVID-19 pandemic, characterized by significant decreases immediately after the pandemic’s onset followed by gradual increases during the pandemic. The initial sharp decrease in our study drug exposure case rates likely reflects the immediate impact of lockdown measures, which could have led to increased supervision of children at home and the reduction in respiratory illnesses, resulting in fewer sales and less use of CC and AA products in the home. Nonprescription sales data from our study showed a large drop in the sale of most nonprescription CC and AA study drugs examined from retail stores to consumers in April 2020, the month after the WHO’s announcement of the pandemic. These factors may account for the significant decline in case rates observed after the public health emergency and may explain why the decrease was more pronounced for CC drugs compared with AA drugs. Even though people were not contracting common seasonal infections, they could still experience other conditions, such as headaches and sprains, necessitating the continued use of analgesics. Parents and caregivers may also have been more vigilant during the early stages of the lockdown and possibly had taken greater precautions to secure medications at home [19–22]. However, as the pandemic continued, this trend reversed, leading to a resurgence in drug exposure case rates, particularly involving unintentional-therapeutic errors. The change to remote schooling and disruptions to daily routines may have increased the risk of drug exposures by providing greater opportunities for children to access medications unsupervised [21, 23, 24].

Our study found distinct age-related patterns in nonprescription CC and AA drug unintentional-general exposures. Among children < 6 years old, there was an immediate sharp drop in unintentional-general exposure case rates involving all CC study drugs, as well as acetaminophen and ibuprofen AA drugs at the onset of the pandemic. Yet, during the pandemic, monthly exposure rates gradually increased and eventually reached pre-pandemic levels approximately 1–1.5 years following the pandemic’s onset. ITS analyses for acetaminophen and ibuprofen AA drugs also revealed that unintentional-general case rates in children < 6 years old statistically significantly decreased in March 2020 and subsequently increased at a statistically significant rate during the pandemic months. Similar trends were observed among pediatric cases of children 6 years of age and greater; however, these findings may be less clinically relevant given that the monthly changes were small. These findings are indicative of age-specific vulnerabilities and behaviors, such as the exploratory nature of younger children and the greater autonomy of older children that might lead to unintended exposures (e.g., a higher dosage than recommended) [25–27].

Our study also showed that unintentional-therapeutic errors involving common CC and AA drugs notably increased from the pre-pandemic to the pandemic period. This shift was most pronounced among children < 6 years old, where unintentional-therapeutic errors sharply decreased at the pandemic’s onset and subsequently increased during the pandemic, reaching or surpassing pre-pandemic rates. These findings highlight a critical need for ongoing support for caregivers in managing medication administration, possibly compounded by disruptions in routine healthcare services which typically serve as touchpoints for reinforcing safe medication practices [2, 9, 26–28].

Historically, the use of certain nonprescription medication has followed seasonal trends, typically peaking during the cold and flu seasons [29, 30]. However, our findings indicate a significant deviation from these patterns during the pandemic. For example, the expected increases in CC drug exposures during the winter months were not observed to the usual extent in the early pandemic period. Instead, there was a sharp decline across all categories immediately following the onset of the pandemic, likely due to the stringent lockdown measures and heightened health awareness that decreased respiratory illnesses [1, 2, 23, 31]. Our drug utilization results showed that sales of the examined nonprescription CC and AA products from retail stores to consumers had similar rise-and-fall patterns as exposure cases. Sales were highest between fall and winter months (September through March), except in the year 2020. However, return to previous nonprescription sales and exposure levels was observed approximately 1.5 years after the pandemic’s onset, despite significant disruptions caused initially.

The increasing trend in unintentional-therapeutic errors involving nonprescription CC and AA medications in our study may reflect the change in healthcare interactions, increase parental stress and depression, or increased reliance on nonprescription medications to avoid exposure to COVID-19, influenza, and other respiratory infections observed during the pandemic [21, 32, 33]. Interestingly, our study did not observe a change in unintentional-therapeutic errors involving prescription statins. Individuals who take these prescription medications, typically for chronic conditions, are likely under the prior care of a healthcare provider. In addition, individuals who use statins are generally older long-term users, often requiring routine blood work and follow-up, unlike individuals who can acquire nonprescription drugs without a healthcare provider’s consult. Unlike nonprescription CC and AA drugs, statins also do not have seasonal patterns of use. Virtual health services and teleconsultations could be strategic components in maintaining continuity of care and supporting safe medication use during similar public health crises.

There are several limitations to our study. Firstly, NPDS includes case data from individuals voluntarily seeking assistance from PCs; case data should not be interpreted as representing the complete incidence of national exposures or cases involving nonprescription CC and AA drugs. Since 2007, there has been a steady decline in PC utilization, likely due to increased reliance on online resources for information [6]. Secondly, NPDS data depend on voluntary caller information. While PCs conduct follow-up on exposure cases, they cannot confirm the accuracy of each case. Drug exposures resulting in unattended or out-of-hospital deaths are unlikely to generate a call to a PC, and thus fatal poisonings are expected to be substantially underreported in NPDS. Also, definitive medical outcomes are not available for all cases owing to unsuccessful follow-up or non-toxic cases not necessitating follow-up. Thus, misclassification of exposure, as a single- versus multiple-product exposure, or misclassification of exposures involving products with more than one CC or AA ingredient versus only one or a few, are concerns given individuals’ awareness of products consumed, familiarity of products among medical providers, and/or discovery of additional information with follow-up. Thirdly, many single-product exposures in our study may have involved multiple active ingredients, since CC products also contain AA ingredients and vice versa; we cannot confidently link exposure cases in our study to a specific single nonprescription CC or AA active ingredient as exposure cases may have been categorized with multiple study drugs. Fourthly, early in the pandemic, news reports stated that the use of non-steroidal anti-inflammatory drugs could worsen COVID-19 infections [34]. On 19 March 2020 the US FDA notified the public that they were not aware of scientific evidence connecting the use of non-steroidal anti-inflammatory drugs with worsening COVID-19 symptoms [35]. Nevertheless, news reports may have led to less ibuprofen use, which could have then resulted in greater reduction of ibuprofen exposures during the pandemic than of other AA drugs [36]. Fifthly, the COVID-19 pandemic might have influenced how these medications were accessed, with shifts in supply and purchasing behaviors, such as stockpiling or supply chain disruptions, potentially causing shortages affecting exposure rates regardless of other factors [37]. Furthermore, ITS analyses were limited by small numbers for some sub-groups; therefore, findings were presented for select nonprescription CC or AA drug categories and the control. Statins as a control group are mostly used in adults and therefore may be more common in households without young children; so, there may be less opportunity for unintentional exposure. Lastly, sales data from both the Consumer Health Insights and PL-ILR databases provide national estimates of units sold for nonprescription drugs from retail stores; however, these data do not capture sales activity such as sales from certain large, national, chain retail stores, the internet, or other specialty stores. Some information, including the active ingredients in some of these nonprescription products, may not be identified or captured from these databases. Therefore, the extent of sales of these products may be underestimated.

## Conclusions

Our study of US PC pediatric unintentional exposures to nonprescription CC and AA drugs observed a marked decrease in exposures at the onset of the pandemic, followed by a gradual rebound over time, with a notable increase in unintentional-therapeutic errors among younger children. These patterns highlight the importance of continuously adapting public health strategies to ensure drug safety during prolonged periods of a public health emergency.

## Supplementary Information

Below is the link to the electronic supplementary material.

Supplementary file1 (PDF 988 KB)

## Declarations

### Data availability

Not applicable. Please see the data source below: America’s Poison Centers maintains the National Poison Data System (NPDS), which houses de-identified records of self-reported information from callers to the country’s poison centers (PCs). NPDS data do not reflect the entire universe of US exposures and incidences related to any substance(s). Exposures do not necessarily represent a poisoning or overdose and America’s Poison Centers are not able to completely verify the accuracy of every report. The views of this article are those of the authors and do not necessarily reflect the opinions of America’s Poison Centers.
