# Supplementary material for: Trends in US Pediatric Unintentional Nonprescription Cold and Cough, Analgesic and Antipyretic Drug Exposure Cases amid the COVID-19 Pandemic
Source: Clin Drug Investig. 2025 May 28;45(7):401–16. doi: 10.1007/s40261-025-01444-9 (PMC12255545; doi:10.1007/s40261-025-01444-9)
Supplement: Supplementary file 1 — Supplementary file1 (PDF 988 KB) [file 40261_2025_1444_MOESM1_ESM.pdf]

Trends in U.S. Pediatric Unintentional Nonprescription Cold and Cough, Analgesic and Antipyretic Drug Exposure Cases Amid the COVID-19 Pandemic

Authors: Sara Karami[1*], Christian Angelo I. Ventura[1*], Ellen Pinnow[1], Jody Green[1], Ajoa Asonye[1], Ibrahim T. Ibrahim[1], Lynda McCulley[1], Gerald Dal Pan[1], Esther H. Zhou[1]

Affiliations:

[1]Center for Drug Evaluation and Research (CDER), U.S. Food and Drug Administration (FDA), Silver Spring, MD, USA

[*]Karami S. and Ventura CAI share dual first authorship.

Corresponding Author:

Esther H. Zhou, Office of Surveillance and Epidemiology, CDER, FDA, 10903 New Hampshire Avenue, Building 22, Room 4212, Silver Spring, MD 20993, [Esther.Zhou@fda.hhs.gov], 301-796-0550

Running title: Pediatric Unintentional OTC Cold/Cough, Analgesic/Antipyretic Exposures Amid the COVID-19 Pandemic

Journal: *Clinical Drug Investigation*, a sister journal of the *Drugs - Real World Outcomes*

Supplementary Material

Fig. 1 Monthly Single-Product Unintentional-General and Unintentional-Therapeutic Error Exposure Case Counts Involving Selected Nonprescription Cold and Cough Drugs by Age Group

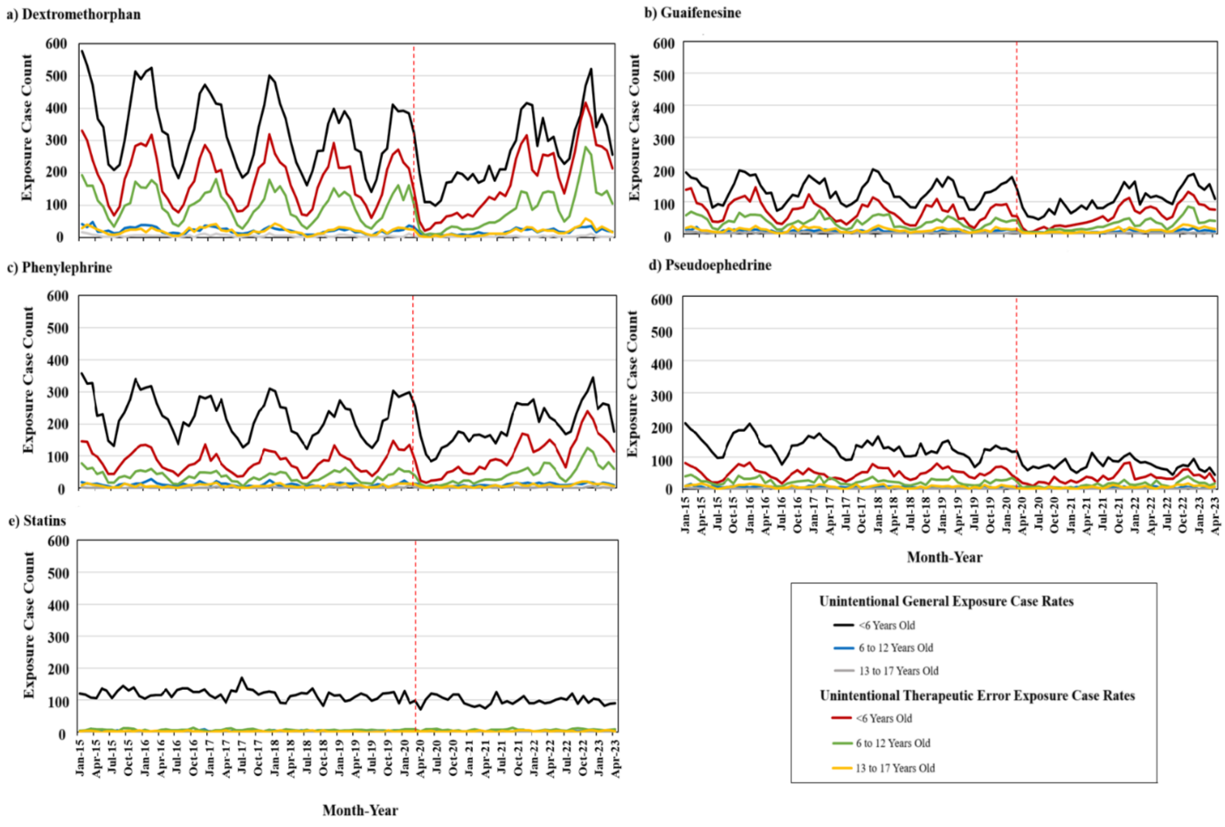

Red vertical line (March 2020) separates study period as pre-pandemic period: January 1, 2015-February 28, 2020; and pandemic period: April 1, 2020-April 30, 2023.

Fig. 2 Monthly Single-Product Unintentional-General and Unintentional-Therapeutic Error Exposure Cases Counts Involving Selected Nonprescription Analgesic and Antipyretic Drugs by Age Group

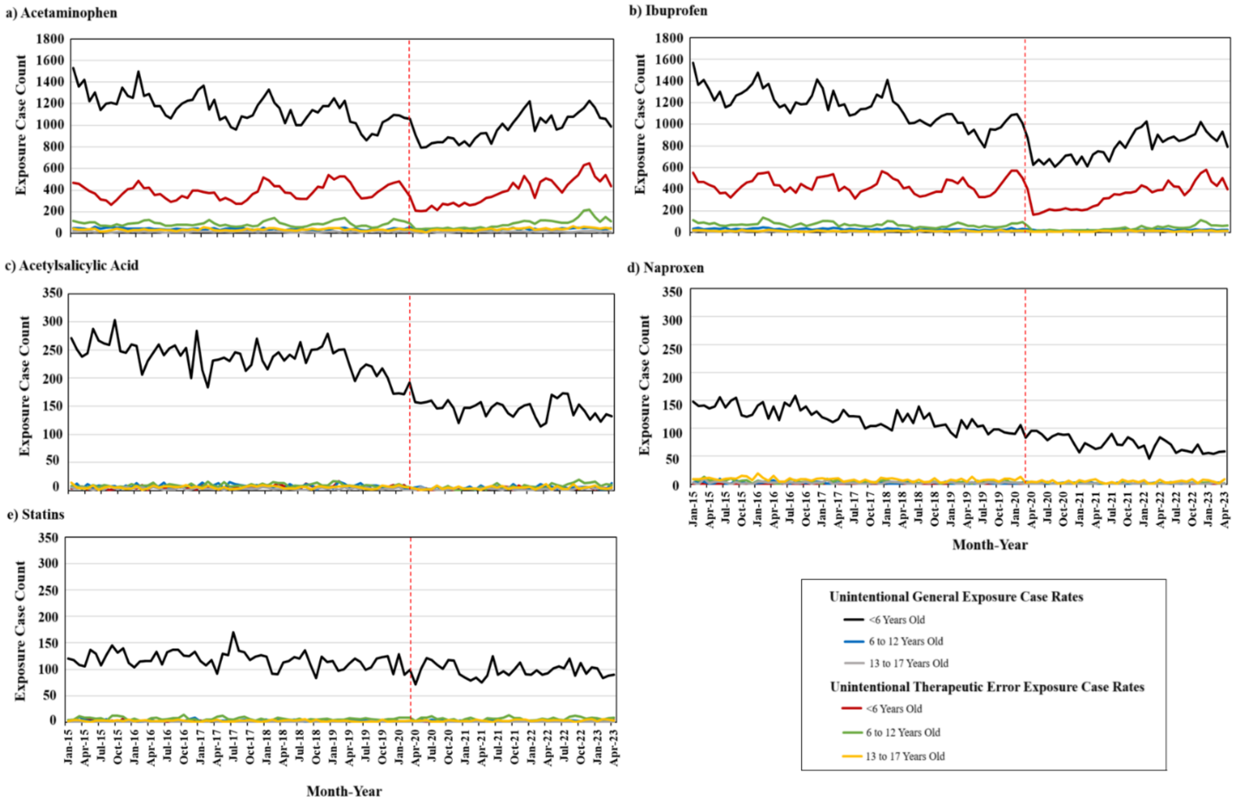

Red vertical line (March 2020) separates study period as pre-pandemic period: January 1, 2015-February 28, 2020; and pandemic period: April 1, 2020-April 30, 2023.
